# Beyond the surface: Exploring differing aspects of wishes to hasten death in patients with amyotrophic lateral sclerosis

**DOI:** 10.1017/S1478951526102673

**Published:** 2026-05-14

**Authors:** Tamara Thurn, Adriano Chiò, Miriam Galvin, Theocharis Stavroulakis, Johanna Anneser

**Affiliations:** 1 Palliative Care/Department of Psychosomatic Medicine and Psychotherapy, School of Medicine and Health, Technical University of Munich, Munich, Germany; 2 Department of Neuroscience “Rita Levi Montalcini”, University of Turin, Turin, Italy; 3 Academic Unit of Neurology, School of Medicine and Global Brain Health Institute, Trinity College Dublin, Dublin, Ireland; 4 Division of Neuroscience, School of Medicine and Population Health, Sheffield Institute for Translational Neuroscience (SITraN), University of Sheffield, Sheffield, UK

**Keywords:** Amyotrophic lateral sclerosis, motor neuron disease, wish to hasten death, wish to die, end of life

## Abstract

**Objectives:**

This study investigates differing aspects of wishes to hasten death (WTHD) distinguished by the extent to which WTHD were linked to patients’ agency: desire for hastened death (DHD), defined as general wishes for death to come sooner, and hastening death intentions (HDI), defined as thoughts about ending one’s life. In particular, this study aims to examine the differences between DHD and HDI in patients with amyotrophic lateral sclerosis (pALS) and identify predictive factors for both.

**Methods:**

A cross-sectional nested study was conducted within a multi-center longitudinal study involving pALS from 5 European countries. Data collected included DHD (Schedule of Attitudes toward Hastened Death), HDI (“could you currently imagine ending your life?”), sociodemographic and clinical characteristics, psychological distress, quality of life, and social and spiritual-existential aspects.

**Results:**

In our sample of 121 pALS, 12.4% (15/121) expressed DHD, and 28.1% (34/121) expressed HDI. Of the 38 patients reporting any WTHD, only 11 experienced both DHD and HDI simultaneously. 23 patients reported HDI without DHD, while 4 patients expressed DHD without HDI. Multivariable logistic regression identified loneliness (OR = 1.33, 95% CI 1.03–1.71, *p* = 0.028) and reduced meaning in life (OR = 0.89, 95% CI 0.84–0.95, *p* < 0.001) as independent predictors of DHD. For HDI, independent predictors were female gender (OR = 3.31, 95% CI 1.37–7.98, *p* = 0.008) and lower spirituality (OR = 0.92, 95% CI 0.88–0.95, *p* < 0.001).

**Significance of results:**

One in 3 pALS expressed WTHD. Our separate analysis of DHD and HDI supports the existence of distinct manifestations of WTHD and varying underlying factors. While DHD and HDI were associated with different predictors, our results point to the crucial role of spiritual-existential factors in the experience of WTHD, identifying these aspects as target points for intervention. This study highlights the importance of a nuanced understanding and communication regarding WTHD.

## Introduction

Amyotrophic lateral sclerosis (ALS) is an incurable, progressive neurodegenerative disease that affects both upper and lower motor neurons, causing spasticity, muscle weakness, and atrophy. Its progression results in functional decline, including difficulties with swallowing and breathing, dysarthria, restricted mobility, and impairments in all activities of daily living. Thus, the illness experience of patients with ALS (pALS) is characterized by increasing dependency and loss of autonomy and control. From diagnosis onward, patients are confronted with the inevitability of death, prompting reflection on personal values, end-of-life preferences, and decisions about life-sustaining measures such as artificial ventilation. As a response to this “uncertain journey towards death” (Ozanne et al. 2013) and the all-encompassing impact of ALS on patients’ lives, wishes to hasten death (WTHD) may emerge during the course of the disease.

WTHD can manifest in different ways, ranging from passive wishes for death to occur sooner to contemplations about actively hastening death, including requests for assisted suicide or euthanasia. Despite vast variations in subjective experiences, underlying motivations, and the clinical and ethical implications raised, most studies do not systematically differentiate between distinct types of WTHD (Erdmann et al. 2021; Rodríguez-Prat et al. 2024). Moreover, in the literature, WTHD are often not clearly distinguished from accepting death, and various terms like “wish to die,” “desire for death,” and “wish to hasten death” have been used synonymously (Monforte-Royo et al. 2012).

WTHD are complex and highly subjective experiences that can fluctuate and change over time (Rosenfeld et al. 2014), may even co-exist with a will to live (Vehling et al. 2018), and have a multifactorial etiology. According to an international consensus definition (Balaguer et al. 2016), WTHD are seen as a reaction to suffering and may emerge in response to one or more domains of suffering, including physical symptoms, psychological distress, existential suffering, or social aspects.

While most research has been conducted in cancer patients (Rodríguez-Prat et al. 2024), some studies have highlighted the specific impact of different disease trajectories on WTHD in patients with life-limiting diseases (Ohnsorge et al. 2019). Studies from the US and the Netherlands show that the proportion of pALS choosing physician-assisted suicide or euthanasia is higher than in cancer patients (Wang et al. 2016; Eenennaam et al. 2023), with pALS reporting more often psychosocial concerns (e.g. anxiety, dependency) and less often physical concerns as reasons for requesting assisted dying (Maessen et al. 2010). Beyond these studies investigating explicit requests for assisted dying as one manifestation of WTHD, others adopted a broader perspective on WTHD in ALS. In these studies, WTHD were associated with illness-related characteristics (e.g. physical function), psychological distress (e.g. depression, reduced quality of life), social concerns (e.g. feeling like a burden), and spiritual-existential aspects (e.g. lower spirituality) (Rabkin et al. 2000, 2015; Stutzki et al. 2014; Verschueren et al. 2019). A recent review on WTHD in ALS (Erdmann et al. 2021) noted that previous studies often lacked a differentiated conceptualization of WTHD and, despite acknowledging its multifactorial etiology, tended to focus on specific aspects of distress as determinants of WTHD. Only a few studies have explored the multidimensional influences on WTHD.

Using nested data of a heterogeneous sample of pALS from a multi-site research project, the purpose of this study was to expand our understanding of the experience of WTHD in ALS. Considering the complex nature of WTHD, this study investigates 2 interrelated but differing aspects of WTHD distinguished by the extent to which wishes for a hastened death were linked to patients’ agency: desire for hastened death (DHD) defined as general wishes for death to come sooner (not necessarily including ideas to contribute actively to its acceleration) and hastening death intentions (HDI) defined as thoughts about ending one’s life – including, but not limited to, concrete suicidal ideations.

In particular, this study aims

1. to examine the prevalence and differences of DHD and HDI in pALS and
2. to explore the multifactorial etiology by examining the influence of physical, psychological, existential, and social factors and comparing the impact of these various factors on DHD and HDI.

## Methods

### Study design and participants

The present study is a nested cross-sectional study within a larger prospective observational multi-site research project on the illness journey of pALS and their caregivers conducted between 2015 and 2019 (ALS CarE – A Program for ALS Care in Europe). The study population comprised a consecutive sample of patients (aged ≥18 years) with a diagnosis of ALS according to the revised El Escorial criteria (Brooks et al. 2000) and regularly scheduled visits at specialist ALS clinics in 5 European countries (Germany, Ireland, Italy, the Netherlands, and the United Kingdom). The treating physicians approached eligible patients during routine visits and informed them about the research project.

**Figure 1.**
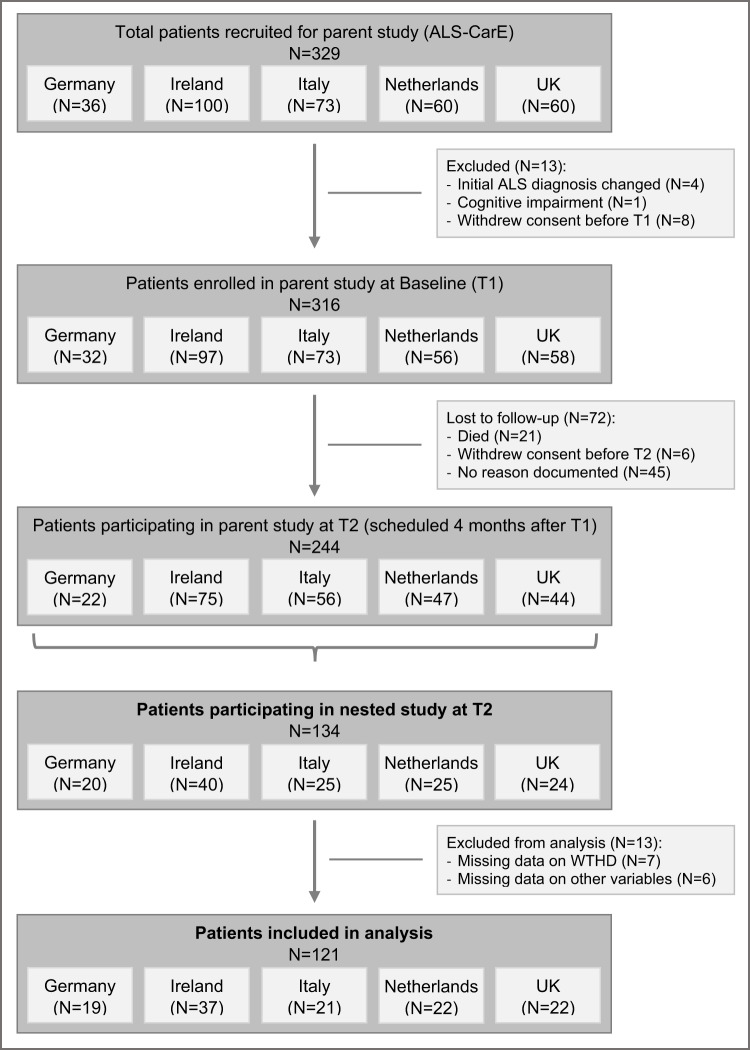
Flow chart of the recruitment process and study participation (for both the parent ALS CarE study and the nested study). Figure 1 long description.

Patients who agreed to participate in the parent study were followed over a course of 18 months with a total of 5 data collection timepoints. At timepoint 2 (scheduled 4 months after study entry), all patients from the parent study were asked to participate in the present nested study focusing on WTHD and existential aspects. Patients who agreed were included in the nested study sample. Figure 1 shows a flow chart of the recruitment process and study participation.

Participation was voluntary and anonymous; informed consent was obtained from all participants. This study was conducted under the ethical guidelines of the Declaration of Helsinki and approved by the institutional review boards of each study site.

### Data collection and measures

Data were obtained through in-person structured interviews by a member of the research team attached to each site. Data on psychological distress, quality of life, and clinical characteristics were gathered on all 5 timepoints of the parent study, while measures on WTHD and spiritual-existential aspects were completed as part of the nested study at timepoint 2 only.

#### Wishes to hasten death (WTHD)

We used 2 approaches to capture interrelated but differing aspects of WTHD (DHD and HDI).

To assess DHD, we used the Schedule of Attitudes toward Hastened Death (SAHD), a validated instrument evaluating the “extent to which patients desire a more rapid death than would occur naturally” (Breitbart et al. 2000). The SAHD consists of 20 items assessing a range of attitudes toward dying (from accepting death to wishes for a sooner death or specific actions toward dying) using a true-false format. Total scores range between 0 and 20; a cut-off of 1 SD above the mean (≥8), as suggested in previous studies (Rosenfeld et al. 1999, 2000), was used to identify patients with a DHD.

To assess HDI (characterized by patients’ agency over hastening death), we used the question “Could you currently imagine ending your life because of your disease?” Answers were captured on an 11-point numeric rating scale (NRS) from 0 (definitely not) to 10 (definitely yes). Analogous to the SAHD, participants were considered as expressing HDI if they had a score of 1 SD above the mean, leading to a cut-off score of ≥7.

#### Psychological distress

The Hospital Anxiety and Depression Scale (HADS) is a 14-item self-report measure of anxiety and depression in people with a physical illness (Zigmond and Snaith 1983). Sum scores (0–21) for each subscale assess the severity of anxiety and depression.

#### Quality of Life (QoL)

The McGill Quality of Life Questionnaire (MQOL) measures patients’ current QoL with 16 items covering different domains of well-being (Cohen et al. 1997). Total scores range from 0 to 10, with higher scores indicating higher QoL.

#### Meaning in Life (MiL)

The Purpose in Life Questionnaire (PIL) is a 20-item attitude scale which quantifies the extent to which respondents experience a sense of purpose and meaning in life (Crumbaugh and Maholick 1964). Total scores range from 20 (low purpose/meaning) to 140 (high purpose/meaning).

#### Spirituality/religiosity

The Functional Assessment of Chronic Illness Therapy – Spiritual Well-Being (FACIT-Sp-12) is a 12-item scale that assesses chronically ill patients’ current spiritual state (Bredle et al. 2011). Item responses are summed to create a total score (0–48) with higher scores reflecting higher levels of spiritual well-being. The Idler Index of Religiosity (Idler 1987) is a 4-item instrument that measures the degree of religiosity from 4 (least religious) to 17 (most religious).

#### Numeric rating scales (NRSs)

NRSs (0–10) were used to cover additional aspects that may be associated with patients’ WTHD, e.g. loneliness, hopelessness, patients’ perception of caregiver burden, and caregiver QoL.

#### Sociodemographic and clinical data

Sociodemographic data comprised age, gender, marital status, and education. Clinical data included time since diagnosis, site of onset, and use of gastrostomy or ventilation. Physical function was assessed with the revised ALS Functional Rating Scale (ALSFRS-R), a well-established instrument for monitoring disease severity, with scores ranging from 0 (locked-in-state) to 48 (no functional impairment) (Cedarbaum et al. 1999). To measure patients’ subjective health status, we used the EQ-VAS (EuroQol Group 1990), a visual analog scale from 0 (worst) to 100 (best).

### Data analysis

Statistical analyses were performed using IBM^®^ Statistical Package for the Social Sciences (SPSS^®^) Version 26.0. All tests were performed with an alpha value set at 0.05. Bonferroni correction for multiple comparisons was performed when relevant.

Descriptive results for both WTHD outcomes were presented as mean ± standard deviations on the score level, and as percentages for categorical outcomes according to the respective cut-offs. To explore the relationship between DHD and HDI, we calculated Spearman correlations and Cohen’s kappa. Further, we compared SAHD responses on the item level between pALS expressing HDI and pALS with no HDI using chi-square tests.

Categorical outcomes for both DHD and HDI were used as dependent variables in all further analyses. In order to identify distinguishing factors for each, analyses were conducted separately for DHD and HDI.

In a 2-step process, we examined the association between the presence of DHD or HDI and the following predictor variables: sociodemographics, physical, psychological, spiritual-existential, and social factors. In a first step, univariable analyses were conducted using Student’s *t*-test for continuous variables and chi-square tests or Fisher’s exact tests for categorical variables. In a second step, 2 multivariable logistic regression analyses were performed to identify variables that provided a unique, statistically significant contribution to predicting DHD or HDI. All variables with a univariable association at a relaxed level of *p* < 0.10 were considered for inclusion in each regression model. Backward elimination was used, excluding the variable with the largest *p*-value first until all *p*-values were smaller than 0.05, to identify the most impactful predictor variables.

Regarding missing data, participants with missing scores on either WTHD outcome variable were excluded from the analysis. For the 20-item SAHD, scores with 4 items or less missing (20%) were considered complete. For missing scores on predictor variables, we used longitudinal data from the parent study to impute missing scores with the row mean.

## Results

### Sample characteristics

A total of 121 pALS (74 male and 47 female) with a mean age of 62.07 years (SD = 10.89) completed the nested study and were included in this data analysis. Table 1 summarizes the sociodemographic and clinical characteristics of our study sample.

**Table 1.** Sociodemographic and clinical characteristics for total Table 1 long description.

	Study sample *N* = 121
	*M* ± SD/*N* (%)
**Sociodemographic characteristics**	
Age (in years)	62.07 ± 10.86 (31–87)
Gender	
Male	74 (61.2%)
Female	47 (38.8%)
Marital status	
Married/partnership	96 (79.4%)
Single	9 (7.4%)
Divorced/separated	11 (9.1%)
Widowed	5 (4.1%)
Education (in years)	13.75 ± 3.84
Employment status	
Paid employment/self-employed	26 (21.5%)
Retired	61 (50.4%)
Unable to work due to illness	25 (20.7%)
Other	4 (3.3%)
Missing	5 (4.1%)
Religion	
Catholic	65 (53.7%)
Protestant	29 (24.0%)
Atheist	26 (21.5%)
Other	1 (0.8%)
**Clinical characteristics**	
Time since diagnosis (in months)	27.20 ± 28.30 (4–162)
Site of onset	
Bulbar	23 (19.0%)
Spinal	92 (76.0%)
Other (respiratory/generalized)	6 (5.0%)
ALSFRS-R total score (0–48)	31.32 ± 9.82
MITOS staging	
Stage 0	52 (43.0%)
Stage 1	28 (23.1%)
Stage 2	18 (14.9%)
Stage 3	3 (2.5%)
Stage 4	2 (1.7%)
Missing	18 (14.9%)
Ventilatory support	
Non-invasive ventilation	37 (30.6%)
Invasive ventilation	-
Enteral nutrition/Gastrostomy	15 (12.4%)

Sample of ALS patients (*N* = 121).

To determine whether selection or missing data may have biased our final sample, *t*-tests and chi-square tests were used to analyze differences between pALS included in the nested study (*N* = 134) and parent study participants not included in the nested study (*N* = 110). We also examined whether participants providing complete data (*N* = 121) differed from those with incomplete data sets (*N* = 13). We found no statistically significant differences between nested study and parent study participants on any sociodemographic, clinical, or psychological variable. Nested study participants with complete data had higher levels of education (*p* = 0.048), higher QoL (*p* = 0.010), and higher MiL (*p* < 0.001) but did not significantly differ from non-completers on WTHD outcomes or any other variable.

### Descriptive data on WTHD in ALS

In our total sample of 121 pALS, the mean SAHD score (DHD) was 4.28 (SD 3.53), and the mean score on the 11-point HDI rating scale was 3.02 (SD 3.98). According to the respective cut-offs, 12.4% of the study sample expressed a DHD (95% CI 6.5–18.3; *N* = 15), and 28.1% (95% CI 20.1–36.1; *N* = 34) expressed HDI. Of those 34 patients expressing HDI, 79.4% (95% CI 65.8–93.0; *N* = 27) indicated that they had already talked about this idea with their caregiver. Combined, 31.4% of our sample (95% CI 23.1–39.7; *N* = 38) reported WTHD on at least one of the 2 outcome measures.

Table 2 presents scores and prevalences for the total sample and for each country separately. In exploratory analysis, both DHD and HDI varied significantly between countries. Post-hoc tests showed that Irish pALS reported lower DHD compared to all other countries (*p*-values between <0.001 and 0.048). With regard to HDI, post-hoc tests indicated that Irish pALS reported lower HDI compared to Italian and Dutch patients (*p* < 0.001/*p* = 0.034). German and UK patients also reported lower HDI than Italian pALS (*p* = 0.01/*p* < 0.001).

**Table 2.** Descriptive results for WTHD in total sample (*N* = 121) and for each country Table 2 long description.

		**Total sample** (*N* = 121)					
		**Germany** (*N* = 19)	**Ireland** (*N* = 37)	**Italy** (*N* = 21)	**Netherlands** (*N* = 22)	**UK** (*N* = 22)	*p*-Value
**DHD** (SAHD score) (0–20; *M* ± SD; range)	4.28 ± 3.53 (0–17)	5.53 ± 4.83 (0–17)	2.11 ± 1.66 (0–7)	4.67 ± 3.97 (0–16)	5.73 ± 3.74 (1–16)	5.05 ± 2.21 (3–12)	<0.001
**DHD** cut-off ≥ 8	15 (12.4%)	4 (21.1%)	0 (0%)	5 (23.8%)	4 (18.2%)	2 (9.1%)	–
**HDI** (NRS score) (0–10; *M* ± SD; range)	3.02 ± 3.98 (0–10)	2.84 ± 4.13 (0–10)	1.30 ± 3.21 (0–10)	6.62 ± 3.46 (0–10)	4.14 ± 3.92 (0–10)	1.55 ± 3.10 (0–10)	<0.001
**HDI** cut-off ≥ 7	34 (28.1%)	4 (21.1%)	5 (13.5%)	14 (66.7%)	9 (40.9%)	2 (9.1%)	–

### Comparison of types of WTHD

On the score level, DHD and HDI were correlated significantly, rho = .514 (*p* < 0.001). When comparing DHD and HDI at the categorical level (Table 3), 83 patients (68.6%) reported neither DHD nor HDI. Of the 38 patients with scores above the cut-off on one or both measures, only 11 scored above on both. Twenty-three patients reported HDI but had an inconspicuous score on the SAHD, while 4 patients expressed DHD without having HDI. Agreement rate between categorical classifications of DHD and HDI was kappa = 0.334 (*p* < 0.001), indicating minimal agreement (McHugh 2012).

**Table 3.** Comparison of both WTHD outcome measures (percentages refer to the total sample, *N* = 121) Table 3 long description.

		desire for hastened death (DHD)	
		No (<8)	Yes (≥8)
**hastening death intentions (HDI)**	No (<7)	83 (68.6%)	4 (3.3%)
	Yes (≥7)	23 (19.0%)	11 (9.1%)

To further explore the relation between DHD and HDI, we examined SAHD responses on the item level. An itemized breakdown of the SAHD (Table 4) shows the extent to which patients differed in their responses depending on their HDI. This table highlights that patients with HDI endorsed SAHD-items related to actions with the intention to end one’s life (items 5, 12, 18, and 20) more frequently than patients with no HDI. They did not differ on items related to options with the possibility of hastening death (i.e. letting disease run its course), wishes to die (without the intention to hasten death), or will to live. Items related to anticipated (physical or emotional) suffering (items 2 and 17) were most frequently endorsed. Anticipated suffering was independent of HDI.

**Table 4.** SAHD item responses stratified by HDI/endorsement of SAHD items and differences in responses between patients with vs without HDI Table 4 long description.

	Total sample (*N* = 121)	No HDI (*N* = 87)	HDI (*N* = 34)	Adjusted *p*-value
SAHD 1: I feel confident that I will be able to cope with the emotional stress of my illness. (F)	9 (8%)	5%	15%	1.000
SAHD 2: I expect to suffer a great deal from emotional problems in the future because of my illness. (T)	66 (56%)	52%	67%	1.000
SAHD 3: My illness has drained me so much that I do not want to go on living. (T)	8 (7%)	3%	15%	0.780
SAHD 4: I am seriously considering asking my doctor for help in ending my life. (T)	13 (11%)	6%	24%	0.140
SAHD 5: Unless my illness improves, I will consider taking steps to end my life. (T)	25 (21%)	12%	46%	**0.020**
SAHD 6: Dying seems like the best way to relieve the pain and discomfort my illness causes. (T)	25 (21%)	15%	36%	0.220
SAHD 7: Despite my illness, my life still has purpose and meaning. (F)	5 (4%)	2%	9%	1.000
SAHD 8: I am careless about my treatment because I want to let the disease run its course. (T)	11 (9%)	7%	16%	1.000
SAHD 9: I want to continue living no matter how much pain or suffering my disease causes. (F)	60 (52%)	45%	70%	0.300
SAHD 10: I hope my disease will progress rapidly because I would prefer to die rather than continue living with this illness. (T)	18 (15%)	15%	15%	1.000
SAHD 11: I have stopped treatment for my illness because I would prefer to let the disease run its course. (T)	4 (3%)	1%	9%	1.000
SAHD 12: I enjoy my present life, even with my illness, and would not consider ending it. (F)	11 (9%)	1%	31%	**0.020**
SAHD 13: Because my illness cannot be cured, I would prefer to die sooner, rather than later. (T)	15 (13%)	7%	27%	0.220
SAHD 14: Dying seems like the best way to relieve the emotional suffering my illness causes. (T)	21 (18%)	13%	29%	0.660
SAHD 15: Doctors will be able to relieve most of the discomfort my illness causes. (F)	38 (33%)	27%	50%	0.360
SAHD 16: Because of my illness, the idea of dying seems comforting. (T)	23 (19%)	14%	33%	0.340
SAHD 17: I expect to suffer a great deal from physical problems in the future because of my illness. (T)	78 (68%)	67%	72%	1.000
SAHD 18: I plan to end my own life when my illness becomes too much to bear. (T)	44 (37%)	25%	68%	**0.020**
SAHD 19: I am aggressively pursuing all possible treatments because I’ll do anything possible to continue living. (F)	28 (24%)	19%	35%	1.000
SAHD 20: I am able to cope with the symptoms of my illness, and have no thoughts of ending my life. (F)	16 (13%)	6%	32%	**0.020**

Item responses indicating WTHD are shown in parentheses (true/false), percentages shown refer to responses indicating WTHD. *p*-Values were adjusted using Bonferroni correction for multiple testing.

### Determinants of DHD and HDI

Table 5 presents the results of univariable analyses to identify factors associated with DHD or HDI.

**Table 5.** Results of univariate analyses: factors associated with WTHD, separate analysis for DHD and HDI Table 5 long description.

	DHD			HDI		
	No DHD (*N* = 106)	DHD ≥8 (*N* = 15)	*p*-Value	No HDI (*N* = 87)	HDI ≥7 (*N* = 34)	*p*-Value
**Sociodemographic characteristics**						
Age (in years; *M* ± SD)	61.90 ± 10.93	63.33 ± 10.55	0.633	62.05 ± 11.39	62.15 ± 9.50	0.964
Female gender	40 (37.7%)	7 (46.7%)	0.507	28 (32.2%)	19 (55.9%)	**0.016**
Married/in a partnership	87 (82.1%)	9 (60.0%)	*0.081*	68 (78.2%)	28 (82.4%)	0.609
Education (in years; *M* ± SD)	13.69 ± 3.91	14.20 ± 3.43	0.315	13.60 ± 3.81	14.12 ± 3.97	0.512
**Physical factors**						
Time since diagnosis (in months)	25.36 ± 26.77	40.20 ± 35.84	*0.057*	25.52 ± 28.46	31.50 ± 27.83	0.298
Site of onset:			0.352			0.594
Bulbar	22 (20.8%)	1 (6.7%)		18 (20.7%)	5 (14.7%)	
Spinal	78 (73.6%)	14 (93.3%)		64 (73.6%)	28 (82.4%)	
Other (respiratory, generalised)	6 (5.7%)	0 (0%)		5 (5.7%)	1 (2.9%)	
Physical function (ALSFRS-R, 0–48)	32.06 ± 9.78	26.13 ± 8.68	**0.028**	32.30 ± 9.98	28.82 ± 9.05	*0.080*
Subjective health (EQ VAS, 0–100)	64.47 ± 22.08	51.67 ± 26.02	**0.042**	63.21 ± 22.77	62.06 ± 23.49	0.805
Ventilation status	34 (32.1%)	3 (20.0%)	0.550	26 (29.9%)	11 (32.4%)	0.791
Gastrostomy status	14 (13.2%)	1 (6.7%)	0.691	12 (13.8%)	3 (8.8%)	0.553
Palliative care status (last 4 months)	14 (13.2%)	3 (20.0%)	0.445	14 (16.3%)	3 (8.8%)	0.390
**Psychological factors**						
Depression (HADS-D score, 0–21)	4.53 ± 3.12	9.40 ± 5.41	**0.004**	4.66 ± 3.25	6.33 ± 4.82	*0.070*
Anxiety (HADS-A score, 0–21)	4.66 ± 3.27	6.51 ± 4.28	*0.051*	4.55 ± 3.17	5.76 ± 3.99	*0.083*
Quality of Life (MQoL 0–10)	6.99 ± 1.26	5.77 ± 1.17	**<0.001**	7.02 ± 1.35	6.38 ± 1.10	**0.015**
**Spiritual-existential factors**						
Religiosity (IIR, 4–17)	8.43 ± 3.94	6.80 ± 3.03	0.126	8.48 ± 4.06	7.58 ± 3.26	0.256
Meaning in Life (PiL, 20–140)	111.30 ± 13.87	86.00 ± 14.77	**<0.001**	111.49 ± 14.92	99.65 ± 16.60	**<0.001**
Spirituality (FACIT-Sp-12, 0–48)	33.21 ± 8.25	21.82 ± 10.55	**<0.001**	33.52 ± 8.98	27.40 ± 8.82	**<0.001**
Hopelessness (NRS, 0–10)	2.80 ± 2.84	5.40 ± 3.76	**0.020**	2.60 ± 2.72	4.47 ± 3.54	**0.008**
**Social factors**						
Loneliness (NRS, 0–10)	1.94 ± 2.28	4.67 ± 3.42	**0.009**	2.06 ± 2.26	2.85 ± 3.27	0.199
Guilt toward caregiver (NRS, 0–10)^a^	4.41 ± 3.74	5.93 ± 3.73	0.156	4.06 ± 3.74	5.88 ± 3.52	**0.016**
Perceived caregiver burden (NRS, 0–10)^a^	6.55 ± 2.95	8.29 ± 2.37	**0.022**	6.35 ± 3.02	7.74 ± 2.48	**0.013**
Perceived caregiver QoL (NRS, 0–10)^a^	7.82 ± 2.07	6.38 ± 1.71	**0.018**	8.03 ± 1.99	6.76 ± 2.03	**0.003**

a Data from *N* < 121 patients because some patients did not have a caregiver – variables were not considered for inclusion in multivariable regression analysis.

All variables with *p* < 0.10 in univariate analysis were considered for inclusion in multivariable analysis.

#### Sociodemographic characteristics

Female patients were more likely to report HDI. There were no statistically significant associations between DHD and sociodemographic characteristics.

#### Physical factors

Patients who expressed DHD reported decreased subjective health and had lower scores on the ALSFRS-R, indicating greater functional impairment. There were no significant associations between HDI and any physical factors.

#### Psychological factors

Both DHD and HDI were significantly associated with reduced QoL. Patients with DHD reported more depressive symptoms. HDI was not significantly associated with depression or anxiety scores.

#### Spiritual-existential factors

Religiosity was not associated with DHD or HDI. Both patients with DHD and patients expressing HDI reported a decreased MiL, lower spirituality, and more hopelessness.

#### Social factors

Both DHD and HDI were significantly associated with higher perceived caregiver burden and lower perceived caregiver QoL. Only DHD was associated with feelings of loneliness, while HDI was associated with feelings of guilt toward caregivers.

#### Multivariable analysis of predictor variables

Following the univariable analyses, 2 separate multivariable logistic regression analyses were conducted to identify the strongest predictors of DHD and HDI. The resulting models are shown in Table 6.

**Table 6.** Results of multivariable logistic regression analysis Table 6 long description.

Predictors	Regression coefficient B (SE)	OR (95% CI)	*p*-Value
***Regression model 1: predicting desire for hastened death (DHD)***			
Loneliness	0.28 (0.13)	1.33 (1.03–1.71)	0.028
Meaning in life (PiL)	−0.11 (0.03)	0.89 (0.84–0.95)	<0.001
***Regression model 2: predicting hastening death intentions (HDI)***			
Female gender	1.20 (0.45)	3.31 (1.37–7.98)	0.008
Spirituality (FACIT-Sp-12)	−0.08 (0.03)	0.92 (0.88–0.97)	<0.001

SE = standard error; OR = odds ratio; CI = confidence interval.

In the regression model for DHD, the only variables remaining as significant predictors were loneliness and MiL. DHD was associated with experiencing loneliness (OR = 1.33, 95% CI 1.03–1.71) and a decreased MiL (OR = 0.89, 95% CI 0.84–0.95). This model was statistically significant (*χ*^2^ = 38.198, *p* < 0.001) and explained 51.3% of variance in DHD (Nagelkerke’s *R*^2^ = .513).

In the separate regression model for HDI, only female gender and spirituality remained as significant predictors. Female patients were 3.3 times more likely to express HDI (OR = 3.31, 95% CI 1.37–7.98). HDI was also associated with lower spirituality (OR = 0.92, 95% CI 0.88–0.95). This model was statistically significant (*χ*^2^ = 18.269, *p* < 0.001) and explained 20.2% of variance in HDI (Nagelkerke’s *R*^2^ = .202).

## Discussion

Nearly one-third of our sample of pALS experienced WTHD. While 12.4% expressed a more general DHD, more patients (28.1%) reported HDI. Previous studies found WTHD rates of 10.5–25% among pALS (Rabkin et al. 2015; Kuzma-Kozakiewicz et al. 2019; Verschueren et al. 2019), and up to 50% when directly assessing interest in assisted dying (Rabkin et al. 2000; Stutzki et al. 2014). Most studies using the SAHD did not report prevalence rates in pALS, yet the mean SAHD score from our sample is comparable to scores reported for cohorts of European and US pALS (Rabkin et al. 2000; Lulé et al. 2014; Andersen et al. 2022) or those reported for advanced cancer patients (Bellido-Pérez et al. 2017).

Of the 38 patients expressing WTHD in our sample, only 11 (28.9%) expressed both DHD and HDI simultaneously. In fact, we found a low agreement between both measures on the categorical level and only moderate correlation on the score level, supporting the assumption that these are interrelated but distinct manifestations of WTHD. Further comparison reveals that, for most patients, experiencing a DHD included ideas about actively hastening death. In contrast, among patients who reported HDI, only 1 out of 3 expressed a pronounced DHD, suggesting that contemplations to actively hasten death can exist independently of other expressions of WTHD and may not always indicate a wish to die. This finding challenges earlier studies depicting a linear progression from acceptance of dying to WTHD and requests for assisted dying (Schroepfer 2006; Granek et al. 2017) and aligns with work proposing a dynamic pattern and co-occurrence of wishes to live and wishes to die or to hasten death (Ohnsorge et al. 2014; Verschueren et al. 2019).

This heterogeneity of WTHD was also reflected in the range of variables associated with DHD and HDI. Univariable analyses showed that DHD was associated with all dimensions investigated, including physical, psychological, spiritual-existential, and social factors. Using multivariable analysis, MiL and loneliness were identified as key determinants for DHD in pALS. Both loss of meaning and loneliness have been recognized as core features of existential distress (Grech and Marks 2017) and have been linked to WTHD in ALS and other patient groups (Stutzki et al. 2014; Belar et al. 2021; Hauswirth et al. 2021). These results support the view of DHD as a response to multidimensional suffering, or rather, above all, a reaction to existential suffering and crisis.

In contrast, HDI was not associated with physical concerns or depression, suggesting that HDI might be less tied to current suffering. Descriptive statistics also hint that DHD was associated with higher levels of distress than HDI. Yet, in this study, we did not directly compare patients with DHD to those reporting HDI. Additionally, we found that most pALS anticipated future (physical and emotional) suffering, yet fear of future suffering did not differentiate between patients expressing HDI and those with no HDI. Multivariable analysis identified female gender and lower spirituality as the strongest predictors of HDI. While previous studies are inconclusive with regard to the influence of gender on WTHD (Erdmann et al. 2021), spirituality has been identified as an important coping resource for pALS (O’Brien and Clark 2015). Patients with higher levels of spirituality reported fewer concerns about death and dying (Murphy et al. 2000) and were less likely to consider assisted suicide (Ganzini et al. 1998). The absence of spirituality – functioning as a stress-buffering and comforting resource – may therefore lead to thoughts about hastening death in the face of a seemingly uncontrollable and burdensome situation. This also links to qualitative research, differentiating between the underlying meanings of WTHD (Nissim et al. 2009; Rodríguez-Prat et al. 2017; Ohnsorge et al. 2019). This line of research suggests that while some WTHD can be seen as a way to escape a burdensome situation and to end suffering, other WTHD might reflect a desire to maintain a sense of control and autonomy.

Furthermore, the explained variance was much lower for HDI (20.2%) than DHD (51.3%), indicating that other aspects relevant to the emergence of HDI may not have been included in our study. While most studies focus on WTHD as a response to suffering, some studies have expanded this perspective by showing that personality factors are linked to requests for assisted suicide (Oldham et al. 2011; Smith et al. 2015). This is not surprising, given that personality traits shape how people react to and cope with stress or illness (Lazarus and Folkman 1984). Future research should aim to explore further why some patients, when faced with uncertainty or (anticipated) suffering, resort to thoughts about actively hastening death while others do not. Such work could contextualize these wishes within patients’ lived experiences, personal values, and habitual patterns of behavior, thought, and emotions.

Overall, our study highlights the (relative) significance of the spiritual-existential domain within the multifactorial etiology of WTHD in ALS. This finding identifies spiritual-existential aspects as potential targets for intervention and stresses the importance of a multiprofessional approach in ALS care. In recent years, psychotherapeutic interventions specifically addressing patients’ sense of meaning and spirituality as a coping resource have been developed and adapted to the ALS setting (Oberstadt et al. 2018; Gould et al. 2024). Results from a controlled trial (Gould et al. 2024) show promising effects on QOL and depression in pALS, but it remains uncertain if these interventions may also affect WTHD.

### Strengths and limitations

This study presents data from a heterogeneous sample of pALS at various stages of the disease trajectory recruited from ALS clinics in 5 European countries. This may strengthen the generalizability of our results. To capture the complexity of WTHD, we assessed 2 aspects of this construct and applied a comprehensive approach to the investigation of determinants for WTHD. No studies we know of have addressed the distinction of agency in WTHD or have systematically examined differing manifestations of WTHD and their underlying factors in ALS.

This work also has several limitations. We cannot exclude the possibility of selection bias since participant recruitment from the parent study was neither randomized nor systematically recorded. Nested study inclusion rates varied widely between study sites and may also contribute to a possible risk for bias. Yet, our preliminary comparison of nested study participants with the parent study cohort showed no systematic differences. Descriptive statistics and exploratory analysis in our study indicate that WTHD outcomes varied between countries, hinting at the influence of sociocultural contexts. However, the high variability in sample sizes and inclusion rates limits the implications that can be drawn from these findings.

Another limitation is that our HDI item had not been piloted or validated. Yet, stratified comparison of the SAHD item response supports its construct validity. It should be noted that the item captures a hypothetical intention; we did not assess if patients already had a specific plan. The wording of the question (“Could you currently imagine ending your own life?”) may seem ambiguous but this was purposively to accommodate legal differences across study sites (for example, euthanasia being a legal end-of-life option only in the Netherlands). Thus, the item captures different ideas about ending life (including but not limited to an interest in assisted dying).

## Conclusion

This study deepens our insight into the heterogeneity of WTHD in general and the experiences of patients living with a progressive neurodegenerative disease in particular. The exploration of distinct manifestations of WTHD has important implications for theoretical understanding and research, ALS patient care, and intervention.

Overall, our results suggest that WTHD, especially HDI, is common among pALS, highlighting the importance of nuanced and open communication about these highly subjective experiences as well as the need for end-of-life conversations acknowledging patients’ individual fears and values while providing information on end-of-life care. Spiritual-existential factors were key determinants of WTHD, identifying loss of meaning, loneliness, and spirituality as target points for psychological interventions in ALS. While DHD was associated with multidimensional aspects of suffering, current and anticipated suffering were insufficient to fully understand why some patients consider actively hastening death, emphasizing the need to look beyond suffering when investigating the underlying mechanism for the emergence of HDI. Further research is needed to explore the relationship between WTHD, especially HDI, and trait constructs and to examine how interpersonal differences in these constructs may shape patients’ responses to illness and suffering.

## 1 Long description

The flowchart outlines the recruitment and participation process for a study. Initially, 329 patients were recruited for the parent study (ALS-CarE) from Germany (36), Ireland (100), Italy (73), Netherlands (60) and UK (60). Thirteen patients were excluded due to changes in ALS diagnosis (4), cognitive impairment (1), or withdrawal of consent before T1 (8), leaving 316 enrolled at Baseline (T1). These included Germany (32), Ireland (97), Italy (73), Netherlands (56) and UK (58). At T2, scheduled four months after T1, 244 patients participated, with losses due to death (21), withdrawal before T2 (6), or undocumented reasons (45). The T2 participants were from Germany (22), Ireland (75), Italy (56), Netherlands (47) and UK (44). For the nested study at T2, 134 patients participated: Germany (20), Ireland (40), Italy (25), Netherlands (25) and UK (24). Thirteen were excluded from analysis due to missing data on WTHD (7) or other variables (6), resulting in 121 included in the final analysis: Germany (19), Ireland (37), Italy (21), Netherlands (22) and UK (22).

Navigate back to 1.

## Table 1 Long description

The table summarizes sociodemographic and clinical characteristics for 121 people with ALS using means with standard deviations (and ranges where given) and counts with percentages. Participants averaged 62.07 years (SD 10.86; range 31–87) and 13.75 years of education (SD 3.84). Gender was 74 male (61.2%) and 47 female (38.8%); most were married or in a partnership (96; 79.4%), with smaller proportions single (7.4%), divorced/separated (9.1%), or widowed (4.1%). Employment status was most often retired (61; 50.4%), followed by paid/self-employment (26; 21.5%) and unable to work due to illness (25; 20.7%); 4.1% were missing. Religion was mainly Catholic (53.7%), then Protestant (24.0%) and atheist (21.5%). Clinically, time since diagnosis averaged 27.20 months (SD 28.30; range 4–162), and onset was predominantly spinal (92; 76.0%) versus bulbar (23; 19.0%). Mean ALSFRS-R total score was 31.32 (SD 9.82) on a 0–48 scale; MITOS staging was concentrated in earlier stages (stage 0: 43.0%; stage 1: 23.1%), with 14.9% missing staging data. Ventilatory support included non-invasive ventilation for 30.6%, no invasive ventilation reported, and enteral nutrition/gastrostomy for 12.4%; missing values in some categories may affect comparisons.

Navigate back to Table 1.

## Table 2 Long description

The table reports two outcomes by country and overall: DHD (SAHD score, 0–20) and HDI (NRS score, 0–10), each shown as mean ± SD with range, plus the number and percent meeting cut-offs (DHD ≥8; HDI ≥7). Overall, mean DHD is 4.28 ± 3.53 (range 0–17) and mean HDI is 3.02 ± 3.98 (0–10), with both outcomes differing across countries (p<.001 for each). For DHD means, the Netherlands is highest at 5.73 ± 3.74 (1–16) and Ireland is lowest at 2.11 ± 1.66 (0–7); Germany 5.53 ± 4.83, Italy 4.67 ± 3.97, and the UK 5.05 ± 2.21. For the DHD cut-off ≥8, 15 of 121 (12.4%) meet it overall; Ireland has 0%, while Italy is highest at 23.8% (5/21), followed by Germany 21.1% (4/19), Netherlands 18.2% (4/22), and UK 9.1% (2/22). For HDI means, Italy is highest at 6.62 ± 3.46 (0–10) and Ireland is lowest at 1.30 ± 3.21 (0–10); Germany 2.84 ± 4.13, Netherlands 4.14 ± 3.92, and UK 1.55 ± 3.10. For the HDI cut-off ≥7, 34 of 121 (28.1%) meet it overall, ranging from 9.1% in the UK (2/22) to 66.7% in Italy (14/21). P-values are provided only for the mean comparisons, and the country sample sizes are small, so percentages may be unstable.

Navigate back to Table 2.

## Table 3 Long description

The table cross-tabulates two binary measures in a sample of 121: desire for hastened death (DHD; no <8, yes ≥8) and hastening death intentions (HDI; no <7, yes ≥7). The largest group had neither DHD nor HDI: 83 participants (68.6%). A smaller group reported DHD without HDI: 23 (19.0%). Few reported HDI without DHD: 4 (3.3%). Eleven participants (9.1%) reported both DHD and HDI. Overall, desire without intention was more common than intention without desire, and most participants fell in the “neither” category.

Navigate back to Table 3.

## Table 4 Long description

The table reports the percentage of respondents endorsing each of 20 SAHD statements in the full sample (N=121) and by HDI status (no HDI N=87; HDI N=34), along with Bonferroni-adjusted p-values for group differences. Endorsement rates were generally higher in the HDI group for items reflecting desire for death or assisted dying, including SAHD 4 (24% vs 6%), SAHD 6 (36% vs 15%), SAHD 13 (27% vs 7%), and SAHD 16 (33% vs 14%), though these were not statistically significant after adjustment. Four items showed significant adjusted differences (p=.020): SAHD 5 “consider taking steps to end my life unless illness improves” (46% HDI vs 12% no HDI), SAHD 18 “plan to end my own life when illness becomes too much” (68% vs 25%), SAHD 12 “enjoy present life and would not consider ending it” (31% vs 1%), and SAHD 20 “able to cope and have no thoughts of ending my life” (32% vs 6%). Several other items had similar endorsement across groups, such as SAHD 10 (15% in both) and SAHD 17 (72% vs 67%). Many adjusted p-values are 1.000, indicating no detectable differences after multiple-testing correction. Percentages reflect endorsement of the response option indicating WTHD, so interpretation depends on each item’s keyed direction (true/false) and should be made cautiously.

Navigate back to Table 4.

## Table 5 Long description

The table reports univariate comparisons of patient characteristics by desire for hastened death (DHD: no DHD, N=87 vs DHD≥7, N=34) and by hastening death intentions (HDI: no HDI, N=106 vs HDI≥8, N=15), with p-values for each factor. For DHD, significant differences show lower physical function (ALSFRS-R 26.13 vs 32.06, p=.028) and worse self-rated health (EQ VAS 51.67 vs 64.47, p=.042), plus higher depression (9.40 vs 4.53, p=.004), lower quality of life (5.77 vs 6.99, p<.001), and markedly lower meaning in life (86.00 vs 111.30, p<.001) and spirituality (21.82 vs 33.21, p<.001). DHD is also associated with higher hopelessness (5.40 vs 2.80, p=.020), loneliness (4.67 vs 1.94, p=.009), higher perceived caregiver burden (8.29 vs 6.55, p=.022), and lower perceived caregiver quality of life (6.38 vs 7.82, p=.018). For HDI, significant associations include female gender (55.9% vs 32.2%, p=.016), lower quality of life (6.38 vs 7.02, p=.015), lower meaning in life (99.65 vs 111.49, p<.001) and spirituality (27.40 vs 33.52, p<.001), higher hopelessness (4.47 vs 2.60, p=.008), and greater caregiver-related distress (guilt p=.016; burden p=.013; caregiver QoL lower, p=.003). Several variables show no clear differences (e.g., age, education, site of onset, ventilation, gastrostomy, recent palliative care), and some are borderline (e.g., time since diagnosis for DHD p=.057; anxiety for DHD p=.051). Results are unadjusted and based on subgroup sizes that vary by analysis, so they indicate associations rather than causation and should be interpreted cautiously, especially for the smaller HDI group.

Navigate back to Table 5.

## Table 6 Long description

The table reports two multivariable logistic regression models with coefficients, odds ratios with 95% confidence intervals, and p-values for predictors of end-of-life outcomes. In model 1 (desire for hastened death), loneliness is associated with higher odds (B=0.28, OR 1.33, 95% CI 1.03–1.71; p=.028), while meaning in life is associated with lower odds (B=−0.11, OR 0.89, 95% CI 0.84–0.95; p<.001). In model 2 (hastening death intentions), female gender is associated with higher odds (B=1.20, OR 3.31, 95% CI 1.37–7.98; p=.008), while spirituality is associated with lower odds (B=−0.08, OR 0.92, 95% CI 0.88–0.97; p<.001). Across both models, protective factors (meaning in life and spirituality) show odds ratios below 1, whereas loneliness and female gender show odds ratios above 1. Confidence intervals for all listed predictors do not cross 1, aligning with the reported statistically significant p-values. Results indicate associations after adjustment for other variables in each model and do not establish causation.

Navigate back to Table 6.
